# Diagnostic accuracy of an in-house Scrub Typhus enzyme linked immunoassay for the detection of IgM and IgG antibodies in Laos

**DOI:** 10.1371/journal.pntd.0008858

**Published:** 2020-12-07

**Authors:** Philip N. D. Elders, Sandhya Dhawan, Ampai Tanganuchitcharnchai, Koukeo Phommasone, Vilada Chansamouth, Nicholas P. J. Day, Jose A. Garcia-Rivera, Jeffrey C. Hertz, Mayfong Mayxay, Manivanh Vongsouvath, Audrey Dubot-Pérès, Matthew T. Robinson, Paul N. Newton, Stuart D. Blacksell

**Affiliations:** 1 Mahidol-Oxford Tropical Research Medicine Unit, Faculty of Tropical Medicine, Mahidol University, Bangkok, Thailand; 2 Lao-Oxford-Mahosot Hospital-Wellcome Trust Research Unit, Microbiology Laboratory, Mahosot Hospital, Vientiane, Lao PDR; 3 Centre for Tropical Medicine and Global Health, Nuffield Department of Medicine, University of Oxford, Oxford, United Kingdom; 4 U.S. Naval Medical Research Unit 2, Phnom Penh, Cambodia; 5 U.S. Naval Medical Research Unit 2, Singapore; 6 Institute of Research and Education Development, University of Health Sciences, Ministry of Health, Vientiane, Lao PDR; 7 Unite des Virus Emergents (UVE: Aix-Marseille Univ–IRD 190 –Inserm 1207 –IHU Mediterranee Infection), Marseille, France; University of Texas at San Antonio John Peace Library, UNITED STATES

## Abstract

Scrub typhus is a major cause of morbidity and mortality in Southeast Asia. Diagnosis of scrub typhus is difficult due to a lack of accessible validated diagnostic tools. Despite its objectivity, the diagnostic accuracy of ELISA tests is influenced by methodological and patient factors. This study aims to evaluate the performance of a novel in-house ELISA developed in the Mahidol Oxford Tropical Medicine Research Unit (MORU) for anti-scrub typhus group IgM and IgG compared to the “gold standard” reference IFA and PCR, and to determine whether the in-house ELISA can be used as a seroepidemiological screening tool and/or stand-alone test for scrub typhus. A total of 1,976 admission and 1,438 participant follow-up sera collected in the Lao PDR (Laos) were tested with ELISA for IgM and IgG. Samples with an ELISA OD≥0.50 were tested with IFA for IgM and/or IgG. A strong positive relationship was present between ELISA ODs and IFA titers for admission IgM (*r*^2^: 0.70, p <0.005) and IgG (*r*^2^: 0.76, p<0.005), and for follow-up IgM and IgG (both *r*^2^: 0.76, p<0.005) samples. The best compromise between sensitivity and specificity for the ELISA OD cut-off is likely to be between 0.8–1.0 for IgM antibodies and 1.2–1.8 for IgG antibodies. These results demonstrate that the diagnostic accuracy of the MORU in-house scrub typhus group ELISA is comparable to that of IFA, with similar results as reported for the commonly used InBios Scrub Typhus Detect ELISA, validating the use of the in-house ELISA. The optimal ELISA cut-off would depend on the use of the test, and the desired sensitivity and specificity. Further studies are required to authenticate the use of these cut-offs in other endemic regions. This in-house ELISA has the potential to replace the imperfect IFA, which could ultimately reduce the burden of scrub typhus by improving the rate of scrub typhus diagnoses in endemic low-resource areas.

## Author summary

Scrub typhus is a mite-borne infectious disease, caused by the *Orientia tsutsugamushi* bacterium, resulting in considerable morbidity and mortality in the Asia-Pacific region. Diagnosis is difficult since it does not present with any distinctive clinical signs compared to other febrile illnesses in this region, except for a necrotic skin lesion (eschar) in some patients. Laboratory tests are therefore crucial to identify scrub typhus. Reliable laboratory tests need significant infrastructure and experienced staff, making access to accurate tests very limited in the low-resource endemic regions. This study aimed to evaluate the accuracy of less expensive and easier-to-use in-house Enzyme Linked Immunoassays (ELISAs) detecting immunoglobulin IgM and IgG antibodies against scrub typhus infection compared to the “gold-standard” Immunofluorescence Assays (IFAs). There was a strong positive relationship between ELISA and IFA antibody levels for both IgM and IgG. If the ELISA would be used as a stand-alone test for scrub typhus diagnosis in Laos, an ideal balance between sensitivity and specificity would likely be around an ELISA optical density (OD) of 0.8–1.0 for IgM and 1.2–1.8 for IgG. There is a need to investigate the use of these cut-offs in other endemic regions, which could improve the rate of scrub typhus diagnosis in low-resource settings.

## Introduction

Scrub typhus is a rickettsial infection caused by a mite-borne bacterium, *Orientia tsutsugamushi*. It is primarily transmitted to humans through bites of infected trombiculid mite larvae, also known as chiggers [1]. Scrub typhus is endemic to rural areas of Southeast Asia, South Asia, East Asia, the Pacific Islands and northern Australia, that was previously known as the “tsutsugamushi triangle” [1,2]. However, the disease has now been detected outside of this area notably the Middle East, Africa and South America [3]. It is a major cause of acute non-malarial febrile illness, and a common neglected cause of morbidity and mortality [4,5]. The clinical manifestations of scrub typhus vary from asymptomatic to severe disease, with complications such as acute renal failure, septic shock, pneumonitis, acute respiratory distress syndrome (ARDS), meningoencephalitis and myocarditis. However, most patients typically present with milder non-specific clinical symptoms, such as fever, headache and rash [1,6]. Given that these clinical manifestations are similar to other acute febrile illnesses in these regions, such as dengue, typhoid, and leptospirosis, the clinical diagnosis of scrub typhus is difficult in the absence of a typical necrotizing skin lesion; an eschar [7,8]. This is further complicated by a lack of awareness of scrub typhus among both physicians and patients and there being a significant lack of accessible and validated diagnostic tools.

The most accurate approach to scrub typhus diagnosis is to use PCR of blood or eschars coupled with well-validated serological techniques [9,10]. However, *O*. *tsutsugamushi* blood PCR is only positive during the initial rickettsaemic phase of the infection and requires significant laboratory infrastructure, which is often not available in the settings where these diseases are most common [9]. This is also the case for *in vitro* culture of the bacteria, for which the necessity of continuous cell lines and experienced staff further limits the usage to specialized facilities [7].

Serological techniques are thus most commonly used to diagnose scrub typhus, as they are relatively simple [8,11]. However, diagnosing rickettsial infections by serology is limited by low sensitivity during the early course of the disease due to high background antibody levels in endemic areas and the requirement for paired samples [11,12]. The current “gold standard” for serological diagnosis is the indirect immunofluorescence assay (IFA). Yet, IFA has several limitations as it is difficult to standardize due to operator subjectivity, it needs appropriate local diagnostic cut-offs, and requires improvement in terms of standardization and ease of use–which restricts its application in areas where scrub typhus is most frequent [7,8,11,13–15].

Given the limitations of other serological methods, the enzyme-linked immunosorbent assay (ELISA) has been extensively evaluated for infectious disease diagnosis and has been found to be reproducible and a reasonably simple test to perform in clinical laboratory settings [7,8,11,14]. The ELISA is relatively standardized and provides an objective optical density (OD) result. Despite its apparent objectivity, the diagnostic accuracy of ELISA tests is influenced by methodological and patient factors, such as the composition of antigenic strains used and variation in levels of background immunity in endemic areas [8,13]. Optimizing the assay by creating region-specific cut-offs to account for differing background antibody levels and cross-reactivity could lead to ELISAs being an accurate, efficient, relatively simple, and affordable alternative to IFA to screen for and possibly diagnose scrub typhus [11,12,16].

This study aims to evaluate the performance of a novel in-house scrub typhus group (STG) ELISA developed in the Mahidol Oxford Tropical Medicine Research Unit (MORU, Bangkok, Thailand) for scrub typhus IgM and IgG using the United States Naval Medical Research Center (NMRC) produced antigens compared to the “gold standard” reference IFA, and PCR, to detect IgM and IgG antibodies, and to determine whether the in-house STG ELISA can be used as a sero-epidemiological screening tool and/or stand-alone test for the diagnosis of scrub typhus without having to follow up with an IFA test.

## Methods

### Ethics statement

Ethical approval was obtained for the fever study from the Lao National Ethics Committee for Health Research (NECHR 026/2014, 27th May 2014) and from the Oxford Tropical Research Ethics Committee (OXTREC 027–14, 19th June 2014). The study protocol was approved by the United States Naval Medical Research Center (NMRC) Institutional Review Board in compliance with all applicable federal regulations governing the protection of human subjects.

#### Sample collection and population inclusion

The sera used were from patients recruited to the Expanded Fever Surveillance (EFS) study in rural Laos aiming to provide a prospective description of the clinical features and aetiology of fever among random selections of outpatients and inpatients attending Salavan, Luang Namtha, and Xieng Khouang Provincial hospitals. Outpatient adults giving informed written consent or children (<15 years), whose parent or guardian gave informed written consent, who presented with a history of fever for ≤8 days and/or admission body temperature ≥38°C, were recruited during the period December 2014—November 2015. From August 2017—January 2018, inpatients of any age giving informed written consent, presenting with history of fever of any duration and/or admission temperature ≥37.5°C (measured as tympanic but corrected to oral), during the study periods were recruited.

Initially, admission sera were tested using both the IgM and IgG ELISA, and PCR. Follow-up sera were aimed to be collected 7–14 days after the admission samples and were tested with both the IgM and IgG ELISA. A minimum OD of 0.50 for ELISA results was used to determine whether a sample should be tested with the “gold standard” IFA to determine the antibody titer. The IFA titer was determined for both the admission and follow-up sample for an antibody isotype if either the admission or follow-up ELISA OD≥0.50 for that antibody isotype. For one participant with an IgM ELISA OD<0.50 for both admission and follow-up sample, the IFA titers for IgM had been determined for unknown reasons but was included in the analysis. To retrospectively validate the ELISA OD cut-off of 0.50, a further 50 samples with ELISA OD results of <0.50 for both admission and follow-up IgM and IgG were retested for both IgM and IgG IFA titers.

#### ELISA

The MORU in-house STG IgM and IgG ELISA uses specific antigens of *O*. *tsutsugamushi* Karp, Kato, Gilliam, and TA716 strains to detect scrub typhus IgM and IgG antibodies, based on the method described in Phanichkrivalkosil et al. (2019) [17]. Briefly, ELISA plates were prepared by coating two U-bottom 96-well microtiter plates with 1) *O*. *tsutsugamushi* strains pooled antigens consisting of Karp, Gilliam, Kato, and TA716 strains and; 2) uninfected (mock) infected cell-lysate antigen in PBS and incubated overnight at 4°C in a humid chamber. Cell-lysate antigens were produced using the same methodology as previously described by Suwanabun et al. (1997) [18]. Serum samples were tested at a 1:100 dilution in 1% skim milk/PBS buffer and 100μl was transferred to each of the *O*. *tsutsugamushi* and mock infected cell-lysate ELISA plates and incubated at 37°C for 1 hour. Following incubation, the plates were washed 4 times with PBS/0.05% Tween 20 solution and bound IgM antibodies were detected by a 30-minute incubation at 37°C with anti-human IgM peroxidase conjugate (Invitrogen Corporation, USA; 1:3,000 dilution, 100μl per well). Following 4 additional washes, the two component tetramethylbenzidine substrate (KPL Inc., Maryland, USA) was mixed in a 1:1 ratio and 100μl added to each well. The plates were then incubated in a dark chamber at room temperature for 30 minutes and 100μl of 1M hydrochloric acid was added to each well to stop the reaction. Plates were read at an optical wavelength of 450nm (minus a reference OD value read at 650nm) with a microtiter plate reader (Thermo Scientific Multiskan FC, Singapore). The ODs from the mock antigen wells were subtracted as background absorbance to give a final average total absorbance (net OD or OD at 450nm). Two negative and two positive control samples were used as a control of assay performance and were included in four wells each on each plate. Control sera were derived from pooled samples from patients in northern Thailand with ELISA net OD < 0.2 (negative control) and ELISA OD > 1.0 (positive control), respectively. The ELISA was considered positive if it provided an OD result of ≥0.50.

#### IFA

IgM and IgG antibodies were detected using IFA slides produced at MORU using *O*. *tsutsugamushi* pooled Karp, Kato, Gilliam whole cell antigens. Participant sera were serially 2-fold diluted from 1:100 to 1:25,600 and the endpoint was determined as the highest titer displaying specific fluorescence. The diagnostic criterion for anti-*O*. *tsutsugamushi* IgM positivity was ≥1:3,200 at admission or follow-up. Criteria for admission and follow-up combined samples to be considered positive were IgM ≥1:3,200 at admission and/or IgM ≥1:3,200 at follow-up with four-fold rise compared to admission. For IgG, the sample was regarded positive if anti-*O*. *tsutsugamushi* IgG was ≥1:1,600 at admission or follow-up. Criteria for admission and follow-up combined samples to be considered positive for IgG, IgG ≥1:1,600 at admission and/or IgG ≥1:1,600 at follow-up with four-fold rise compared to admission [19].

#### PCR

Nucleic acids were extracted from admission EDTA buffy coat. A probe based real-time PCR assay was used to detect *O*. *tsutsugamushi* (47kDa *htra* gene) [20].

#### Analysis

To evaluate the performance of the MORU in-house STG IgM and IgG ELISA compared to the reference IFA to detect antibodies, the relationship between the ELISA and IFA was determined by comparing the ELISA OD results and the IFA endpoint titers for admission and follow-up samples using Spearman’s correlation coefficient.

To evaluate the suitability of the in-house STG IgM and IgG ELISA for cross-sectional seroprevalence studies or as a stand-alone diagnostic test, a range of IgM and IgG ELISA cut-offs with steps of 0.25 were compared by calculating the sensitivity, specificity and Area Under Receiver Operator Characteristic curve (AUROCC) for the samples compared to IFA positivity and PCR positivity. To more precisely examine the potential cut-off for ELISA OD compared to IFA; sensitivity, specificity and AUROCC was calculated using steps of 0.1 ELISA ODs around the plateau, which ranged from 0.5–1.5 for IgM and 1.0–2.0 for IgG. To examine the sensitivity and specificity of IgG ELISA OD ≥0.5 cut-off used in background antibody seroprevalence screening studies [11], the sensitivity and specificity compared to an IFA titer of 1:100 was calculated. Statistical analyses were performed using STATA 15.1 (StataCorp, College Station, TX).

## Results

### Participant characteristics and number of tests

Sera from 1,976 participants were included; 52% of participants were male. The median age of the participants was 22 years (Interquartile range (IQR): 7–41 years) with a range from 7 days to 90 years, and a total of 835 children <18 years. The median days of illness at participant inclusion was 4 days (IQR: 2–6 days, n = 1,965).

A total of 1,976 admission and 1,438 follow-up samples were tested with ELISA for IgM and IgG ODs (S1 Fig shows the frequency distribution of ELISA ODs). The median interval between admission and follow up sera was 14 days (IQR: 8–20 days, n = 1,437). Of these participants, 310 admission and 276 follow-up samples were tested for IgM IFA titers and 545 admission and 454 follow-up samples for IgG IFA titers (S1 and S2 Tables). A total of 1,939 of the admission samples were tested via PCR.

### ELISA OD cut-off and STG positivity

An ELISA OD ≥0.5 was used as the cut-off in this study to decide whether samples would be tested by IFA. All samples with an ELISA OD <0.5 that were tested with IFA (admission IgM n = 143, follow-up IgM n = 88, admission IgG n = 110, follow-up IgG n = 86) had an IFA titer ≤1:800 and thus none was considered positive for STG (S1 Table). The IFA titers of samples with ELISA OD≥0.5 are presented in S2 Table. Of the IgM samples with an ELISA OD≥0.5, a total of 17/167 (10.2%) of the admission, 31/188 (16.4%) of the follow-up and 35/257 (13.6%) of combined samples tested positive for STG using IFA (Table 1). For the IgG samples with an ELISA OD≥0.5, 51/435 (11.7%) of the admission, 66/368 (17.9%) of the follow-up and 77/495 (15.6%) of the combined samples tested positive for STG using IFA (Table 1). If IgM and/or IgG had an ELISA OD≥0.5, a total of 67/533 (12.6%) of admission samples, 90/471 (19.1%) of follow-up samples and 105/632 (16.6%) of combined was positive using IFA for IgM and/or IgG.

**Table 1:** Number of participants tested for IgM and IgG antibodies with ELISA and/or IFA compared to the number of participants with ELISA Optical Density (OD) ≥0.50 or positive PCR.

Sample type and ELISA Test	Total number of participants screened with ELISA	Number of IFA positive* / number of ELISA OD≥0.50 (%)	Number of ELISA OD≥0.50 / number of PCR positive^	Number of IFA positive* / number of PCR positive^
**IgM**				
Admission	1976	17/167 (10.2%)	9/20	4/13
Follow-up	1438	31/188 (16.4%)	11/15	7/11
Combined	n.a.	35/257 (13.6%)	13/20	9/13
**IgG**				
Admission	1976	51/435 (11.7%)	10/20	1/15
Follow-up	1438	66/368 (17.9%)	12/15	6/12
Combined	n.a.	77/495 (15.6%)	15/20	6/15
**IgM & IgG**†				
Admission	1976	67/533 (12.6%)	16/20	5/19
Follow-up	1438	90/471 (19.1%)	15/15	11/15
Combined	n.a.	105/632 (16.6%)	19/20	13/19

* IFA was considered positive for admission and follow-up if titer was IgM ≥1:3,200; IgG ≥1:1,600. For both admission and follow-up samples combined if IFA titer was IgM ≥1:3,200 at admission and/or IgM ≥1:3,200 at follow-up with four-fold rise compared to admission; IgG ≥1:1,600 at admission and/or IgG ≥1:1,600 at follow-up with four-fold rise compared to admission.

^ PCR was determined on admission sample for 1939 samples. 15/20 positive PCR samples had follow-up ELISA available.

**†** Was deemed positive if IgM and/or IgG was positive

### PCR results

One percent (20/1939) of the samples tested positive with PCR, of which 15 participants had both admission and follow-up sera collected. See S3 Table for an overview of participant characteristics, ELISA OD and IFA titer of these participants. A total of 16/20 (80.0%) positive PCR samples had an ELISA OD≥0.5 at admission for IgM and/or IgG and 19/20 (95.0%) PCR samples had an ELISA OD≥0.5 for either admission or follow-up IgM or IgG. The positive PCR sample that did not have a single ELISA OD≥0.5 did not have a follow-up sample available. All participants with an available follow-up sample and a positive PCR at admission had an ELISA OD≥0.5 for either IgM or IgG at follow-up (15/15).

### Direct comparison of ELISA with IFA results

**Fig 1:**
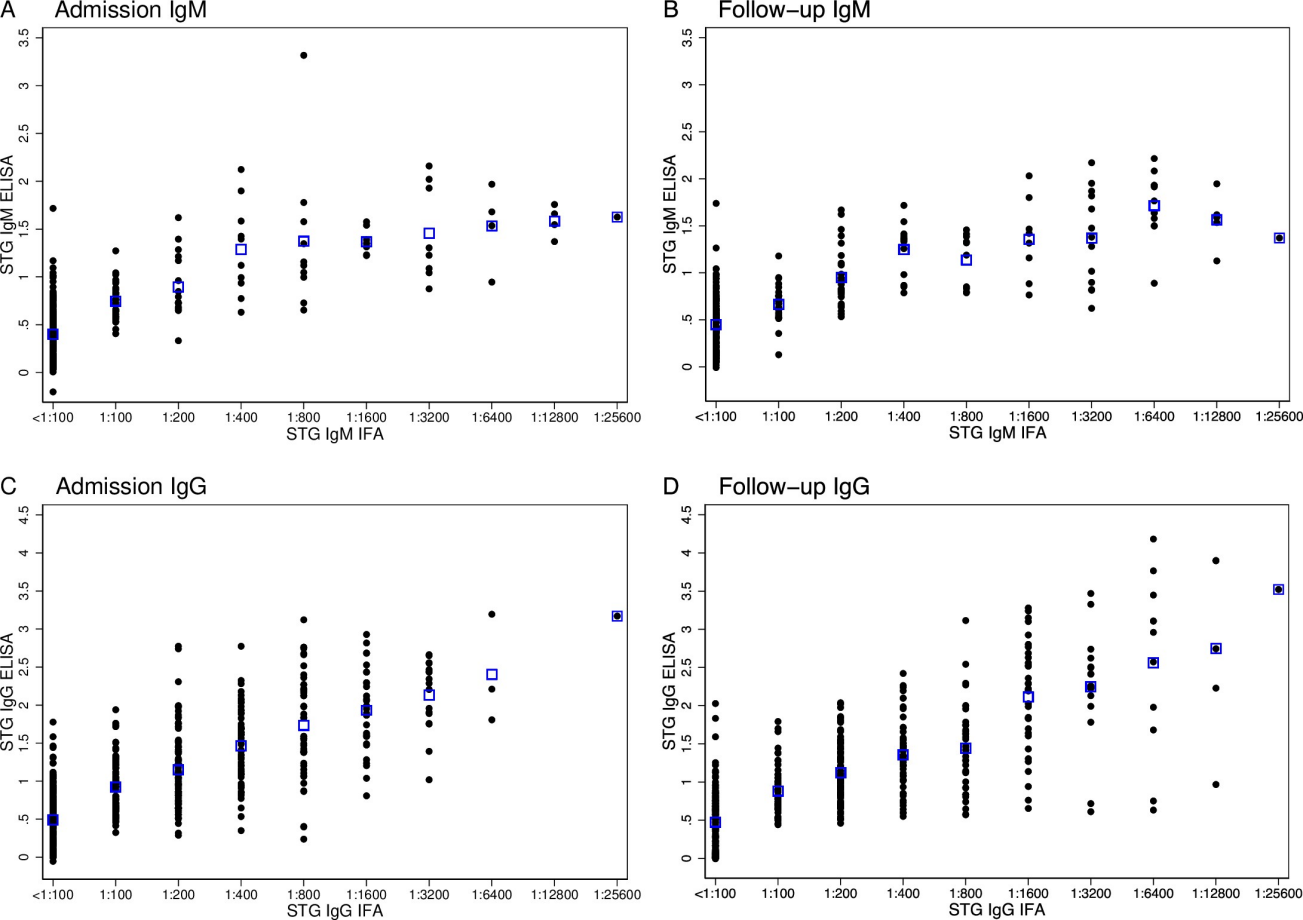
Distribution of ELISA Optical Densities (ODs) compared to IFA titers for admission IgM (A), follow-up IgM (B) admission IgG (C) and follow-up IgG (D). Blue boxes represent the mean ELISA OD for each respective IFA titer.

A strong positive association is present between ELISA ODs and IFA titers for admission IgM (*r*^2^ = 0.70, p<0.005), follow-up IgM (*r*^2^ = 0.76, p<0.005), and for admission and follow-up IgG (both *r*^2^ = 0.76, p<0.005) samples (Fig 1).

### Cut-off ELISA ODs compared against IFA and PCR STG-positive confirmed samples

**Fig 2:**
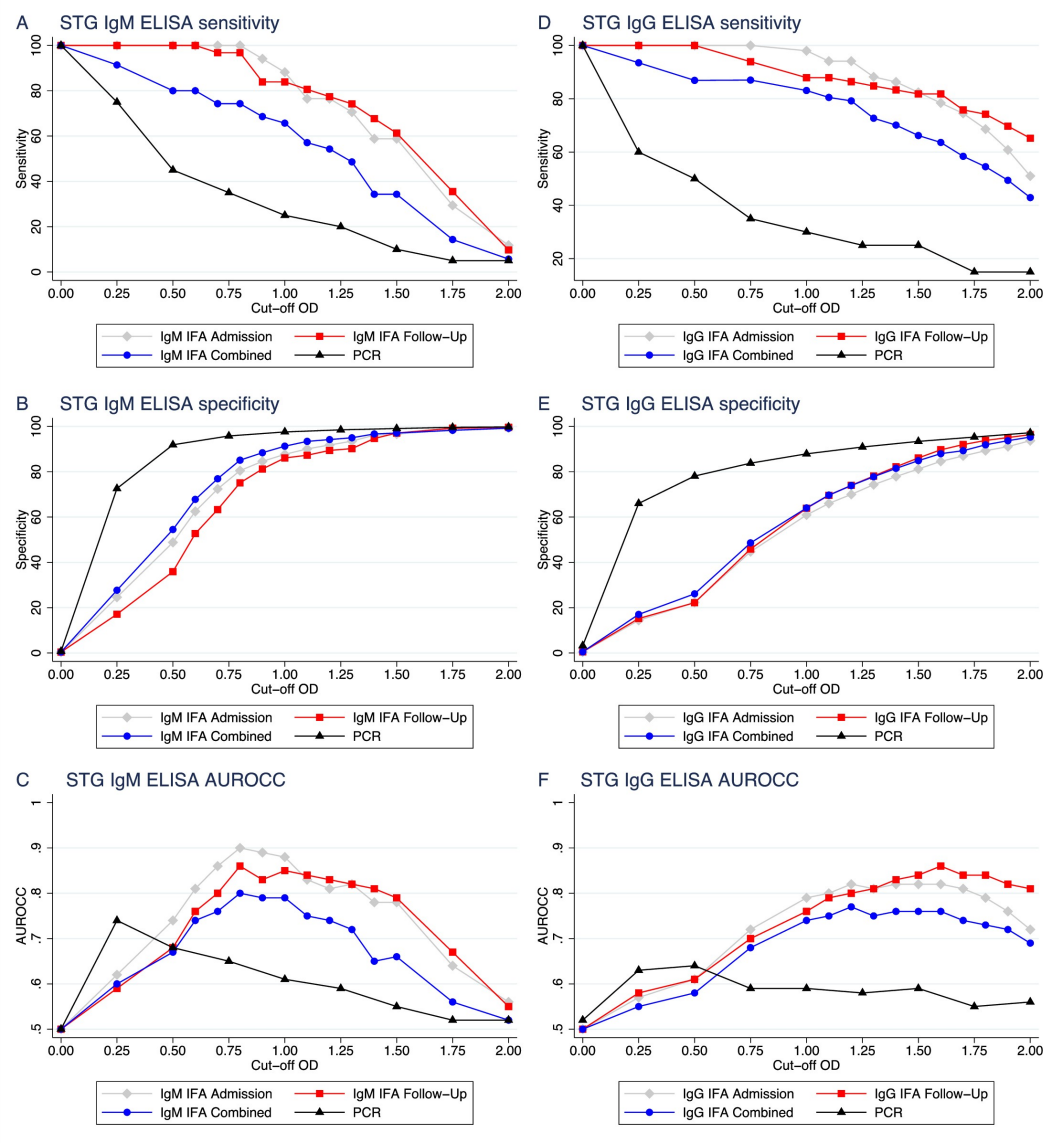
Sensitivity, specificity and Area Under Receiver Operator Characteristic Curve (AUROCC) plots for a range of ELISA cut-off optical densities (ODs) against the different diagnostic modalities. Admission ELISA compared to admission IFA ◆, follow-up ELISA compared to follow-up IFA ■, admission ELISA compared to combined IFA ●, and admission ELISA compared to admission PCR ▲ for IgM (A-C) and IgG (D-F).

A range of cut-off ODs for the ELISA results were compared to positive IFA titers and positive PCR results, which are depicted in Fig 2 with the corresponding sensitivity, specificity and AUROCC in Table 2.

**Table 2:** Sensitivity, specificity and Area Under Receiver Operator Characteristic Curve (AUROCC) for admission, follow-up and combined samples for a range of diagnostic cut-off values of the in-house IgM and IgG ELISA compared against a positive IgM or IgG IFA titers* and PCR.

IgM	Cut-off OD	Sensitivity % (95% CI)	Specificity % (95% CI)	AUROCC (95% CI)
Admission ELISA OD against positive admission IgM IFA	≥0.00	100.0 (80.5–100.0)	0.3 (0.0–1.9)	0.50 (0.50–0.51)
	≥0.25	100.0 (80.5–100.0)	24.6 (19.8–29.9)	0.62 (0.60–0.65)
	≥0.50	100.0 (80.5–100.0)	48.8 (42.9–54.7)	0.74 (0.72–0.77)
	≥0.60	100.0 (80.5–100.0)	62.5 (56.6–68.0)	0.81 (0.78–0.84)
	≥0.70	100.0 (80.5–100.0)	72.4 (66.9–77.4)	0.86 (0.84–0.89)
	≥0.80	100.0 (80.5–100.0)	80.5 (75.5–84.9)	0.90 (0.88–0.93)
	≥0.90	94.1 (71.3–99.9)	84.6 (80.0–88.6)	0.89 (0.83–0.96)
	≥1.00	88.2 (63.6–98.5)	87.7 (83.4–91.2)	0.88 (0.80–0.96)
	≥1.10	76.5 (50.1–93.2)	90.1 (86.1–93.3)	0.83 (0.73–0.94)
	≥1.20	76.5 (50.1–93.2)	91.8 (88.1–94.7)	0.81 (0.74–0.95)
	≥1.30	70.6 (44.0–89.7)	93.5 (90.1–96.1)	0.82 (0.71–0.93)
	≥1.40	58.8 (32.9–81.6)	96.2 (93.4–98.1)	0.78 (0.65–0.90)
	≥1.50	58.8 (32.9–81.6)	96.6 (93.8–98.4)	0.78 (0.66–0.90)
	≥1.75	29.4 (10.3–56.0)	98.6 (96.5–99.6)	0.64 (0.53–0.75)
	≥2.00	11.8 (1.5–36.4)	99.3 (97.6–99.9)	0.56 (0.48–0.63)
Follow-up ELISA OD against positive follow-up IFA	≥0.00	100.0 (88.8–100.0)	0.4 (0.0–2.3)	0.50 (0.50–0.51)
	≥0.25	100.0 (88.8–100.0)	17.1 (12.6–22.5)	0.59 (0.56–0.61)
	≥0.50	100.0 (88.8–100.0)	35.9 (29.9–42.3)	0.68 (0.65–0.71)
	≥0.60	100.0 (88.8–100.0)	52.7 (46.2–59.0)	0.76 (0.73–0.79)
	≥0.70	96.8 (83.3–99.9)	63.3 (56.9–69.3)	0.80 (0.76–0.84)
	≥0.80	96.8 (83.3–99.9)	75.1 (69.2–80.4)	0.86 (0.82–0.90)
	≥0.90	83.9 (66.3–94.5)	81.2 (75.8–85.9)	0.83 (0.76–0.90)
	≥1.00	83.9 (66.3–94.5)	86.1 (81.2–90.2)	0.85 (0.78–0.92)
	≥1.10	80.6 (62.5–92.5)	87.3 (82.5–91.2)	0.84 (0.77–0.91)
	≥1.20	77.4 (58.9–90.4)	89.4 (84.8–92.9)	0.83 (0.76–0.91)
	≥1.30	74.2 (55.4–88.1)	90.2 (85.8–93.6)	0.82 (0.74–0.90)
	≥1.40	67.7 (48.6–83.3)	94.7 (91.1–97.1)	0.81 (0.73–0.90)
	≥1.50	61.3 (42.2–78.2)	97.1 (94.2–98.8)	0.79 (0.70–0.88)
	≥1.75	35.5 (19.2–54.6)	99.2 (97.1–99.9)	0.67 (0.59–0.76)
	≥2.00	9.7 (2.0–25.8)	99.6 (97.7–100.0)	0.55 (0.49–0.60)
Admission ELISA OD against positive admission or follow-up IFA	≥0.00	100.0 (90.0–100.0)	0.4 (0.0–2.3)	0.50 (0.50–0.51)
	≥0.25	91.4 (76.9–98.2)	27.7 (22.1–33.8)	0.60 (0.54–0.65)
	≥0.50	80.0 (63.1–91.6)	54.5 (48.0–60.9)	0.67 (0.60–0.75)
	≥0.60	80.0 (63.1–91.6)	67.8 (61.5–73.6)	0.74 (0.67–0.81)
	≥0.70	74.3 (56.7–87.5)	76.9 (71.0–82.0)	0.76 (0.68–0.83)
	≥0.80	74.3 (56.7–87.5)	85.1 (80.0–89.4)	0.80 (0.72–0.87)
	≥0.90	68.6 (50.7–83.1)	88.4 (83.7–92.2)	0.79 (0.70–0.87)
	≥1.00	65.7 (47.8–80.9)	91.3 (87.0–94.5)	0.79 (0.70–0.87)
	≥1.10	57.1 (39.4–73.7)	93.4 (89.5–96.2)	0.75 (0.67–0.84)
	≥1.20	54.3 (36.6–71.2)	94.2 (90.5–96.8)	0.74 (0.66–0.83)
	≥1.30	48.6 (31.4–66.0)	95.0 (91.5–97.4)	0.72 (0.63–0.80)
	≥1.40	34.3 (19.1–52.2)	96.7 (93.6–98.6)	0.65 (0.57–0.74)
	≥1.50	34.3 (19.1–52.2)	97.1 (94.1–98.8)	0.66 (0.58–0.74)
	≥1.75	14.3 (4.8–30.3)	98.3 (95.8–99.5)	0.56 (0.50–0.62)
	≥2.00	5.7 (0.7–19.2)	99.2 (97.0–99.9)	0.52 (0.49–0.56)
Admission ELISA OD against positive PCR	≥0.00	100.0 (83.2–100.0)	0.7 (0.4–1.2)	0.50 (0.50–0.51)
	≥0.25	75.0 (50.9–91.3)	72.6 (70.5–74.6)	0.74 (0.64–0.84)
	≥0.50	45.0 (23.1–68.5)	91.9 (90.6–93.1)	0.68 (0.57–0.80)
	≥0.75	35.0 (15.4–59.2)	95.8 (94.8–96.6)	0.65 (0.55–0.76)
	≥1.00	25.0 (8.7–49.1)	97.6 (96.8–98.2)	0.61 (0.52–0.71)
	≥1.25	20.0 (5.7–43.7)	98.5 (97.8–99.0)	0.59 (0.50–0.68)
	≥1.50	10.0 (1.2–31.7)	99.1 (98.5–99.4)	0.55 (0.48–0.61)
	≥1.75	5.0 (0.1–24.9)	99.6 (99.2–99.8)	0.52 (0.47–0.57)
	≥2.00	5.0 (0.1–24.9)	99.8 (99.5–100.0)	0.52 (0.48–0.57)
IgG	Cut-off OD	Sensitivity % (95% CI)	Specificity % (95% CI)	AUROCC (95% CI)
Admission ELISA OD against positive admission IgG IFA	≥0.00	100.0 (93.0–100.0)	0.4 (0.0–1.5)	0.50 (0.50–0.50)
	≥0.25	100.0 (93.0–100.0)	14.4 (11.4–17.8)	0.57 (0.56–0.59)
	≥0.50	100.0 (93.0–100.0)	22.3 (18.7–26.2)	0.61 (0.59–0.63)
	≥0.75	100.0 (93.0–100.0)	44.7 (40.3–49.2)	0.72 (0.70–0.75)
	≥1.00	98.0 (89.6–100.0)	60.9 (56.5–65.3)	0.79 (0.77–0.82)
	≥1.10	94.1 (83.3–98.8)	66.0 (61.6–70.2)	0.80 (0.76–0.84)
	≥1.20	94.1 (83.3–98.8)	70.0 (65.8–74.1)	0.82 (0.78–0.86)
	≥1.30	88.2 (76.1–95.6)	74.3 (70.2–78.1)	0.81 (0.76–0.86)
	≥1.40	86.3 (73.7–94.3)	77.9 (74.0–81.5)	0.82 (0.77–0.87)
	≥1.50	82.4 (69.1–91.6)	81.2 (77.4–84.5)	0.82 (0.76–0.87)
	≥1.60	78.4 (64.7–88.7)	84.6 (81.1–87.7)	0.82 (0.76–0.87)
	≥1.70	74.5 (60.4–85.7)	87.0 (83.8–89.9)	0.81 (0.75–0.87)
	≥1.80	68.6 (54.1–80.9)	89.3 (86.2–91.9)	0.79 (0.72–0.86)
	≥1.90	60.8 (46.1–74.2)	91.1 (88.2–93.5)	0.76 (0.69–0.83)
	≥2.00	51.0 (36.6–65.2)	93.7 (91.2–95.7)	0.72 (0.65–0.79)
Follow-up ELISA OD against positive follow-up IFA	≥0.00	100.0 (94.6–100.0)	0.5 (0.1–1.8)	0.50 (0.50–0.51)
	≥0.25	100.0 (94.6–100.0)	15.2 (11.8–19.2)	0.58 (0.56–0.59)
	≥0.50	100.0 (94.6–100.0)	22.2 (18.1–26.6)	0.61 (0.59–0.63)
	≥0.75	93.9 (85.2–98.3)	45.9 (40.8–51.0)	0.70 (0.66–0.74)
	≥1.00	87.9 (77.5–94.6)	63.9 (58.9–68.7)	0.76 (0.71–0.81)
	≥1.10	87.9 (77.5–94.6)	69.6 (64.7–74.1)	0.79 (0.74–0.83)
	≥1.20	86.4 (75.7–93.6)	74.0 (69.3–78.3)	0.80 (0.75–0.85)
	≥1.30	84.8 (73.9–92.5)	78.1 (73.6–82.1)	0.81 (0.77–0.86)
	≥1.40	83.3 (72.1–91.4)	82.2 (78.0–85.9)	0.83 (0.78–0.88)
	≥1.50	81.8 (70.4–90.2)	86.1 (82.2–89.4)	0.84 (0.79–0.89)
	≥1.60	81.8 (70.4–90.2)	89.7 (86.2–92.5)	0.86 (0.81–0.91)
	≥1.70	75.8 (63.6–85.5)	92.0 (88.9–94.5)	0.84 (0.79–0.89)
	≥1.80	74.2 (62.0–84.2)	93.8 (90.9–96.0)	0.84 (0.79–0.89)
	≥1.90	69.7 (57.1–80.4)	95.1 (92.5–97.0)	0.82 (0.77–0.88)
	≥2.00	65.2 (52.4–76.5)	96.4 (94.0–98.0)	0.81 (0.75–0.87)
Admission ELISA OD against positive admission or follow-up IFA	≥0.00	100.0 (95.3–100.0)	0.5 (0.1–1.9)	0.50 (0.50–0.51)
	≥0.25	93.5 (85.5–97.7)	17.0 (13.3–21.1)	0.55 (0.52–0.59)
	≥0.50	86.9 (80.6–95.4)	26.1 (21.8–30.8)	0.58 (0.54–0.62)
	≥0.75	87.0 (77.4–93.6)	48.6 (43.5–53.7)	0.68 (0.63–0.72)
	≥1.00	83.1 (72.9–90.7)	64.0 (58.9–68.8)	0.74 (0.69–0.78)
	≥1.10	80.5 (69.9–88.7)	69.7 (64.8–74.3)	0.75 (0.70–0.80)
	≥1.20	79.2 (68.5–87.6)	73.9 (69.2–78.2)	0.77 (0.71–0.82)
	≥1.30	72.7 (61.4–82.3)	77.8 (73.3–81.9)	0.75 (0.70–0.81)
	≥1.40	70.1 (58.6–80.0)	81.5 (77.2–85.2)	0.76 (0.70–0.81)
	≥1.50	66.2 (54.6–76.6)	84.9 (80.9–88.3)	0.76 (0.70–0.81)
	≥1.60	63.6 (51.9–74.3)	88.0 (84.3–91.1)	0.76 (0.70–0.81)
	≥1.70	58.4 (46.6–69.9)	89.3 (85.8–92.2)	0.74 (0.68–0.80)
	≥1.80	54.5 (42.8–65.9)	91.9 (88.7–94.4)	0.73 (0.67–0.79)
	≥1.90	49.4 (37.8–61.0)	93.7 (90.8–95.9)	0.72 (0.66–0.77)
	≥2.00	42.9 (31.6–54.6)	95.3 (92.7–97.2)	0.69 (0.63–0.75)
Admission ELISA OD against positive PCR	≥0.00	100.0 (83.2–100.0)	3.2 (2.5–4.1)	0.52 (0.51–0.52)
	≥0.25	60.0 (36.1–80.9)	66.0 (63.9–68.1)	0.63 (0.52–0.74)
	≥0.50	50.0 (27.2–72.8)	78.1 (76.1–79.9)	0.64 (0.53–0.75)
	≥0.75	35.0 (15.4–59.2)	83.8 (82.1–85.4)	0.59 (0.49–0.70)
	≥1.00	30.0 (11.9–54.3)	87.9 (86.4–89.3)	0.59 (0.49–0.69)
	≥1.25	25.0 (8.7–49.1)	90.9 (89.6–92.2)	0.58 (0.48–0.68)
	≥1.50	25.0 (8.7–49.1)	93.4 (92.2–94.5)	0.59 (0.49–0.69)
	≥1.75	15.0 (3.2–37.9)	95.3 (94.3–96.2)	0.55 (0.47–0.63)
	≥2.00	15.0 (3.2–37.9)	97.2 (96.4–97.9)	0.56 (0.48–0.64)

* IFA was considered positive for admission and follow-up if titer was IgM ≥1:3,200; IgG ≥1:1,600. For combined samples if IFA titer was IgM ≥1:3,200 at admission and/or IgM ≥1:3,200 at follow-up with four-fold rise compared to admission; IgG ≥1:1,600 at admission and/or IgG ≥1:1,600 at follow-up with four-fold rise compared to admission. The combined IFA samples are compared against the admission ELISA. The shaded sections indicate the ELISA cut-offs with the highest AUROCC.

The best compromise for the ELISA OD cut-off for acute scrub typhus diagnosis with IgM antibodies was likely to be in the range of 0.8–1.0 with sensitivity, specificity and AUROCC for ELISA OD at ≥1.0 of 88.2%, 87.7%, and 0.88 for admission, 83.9%, 86.1%, and 0.85 for follow-up and of 65.7%, 91.3%, and 0.79 for combined samples. Whilst for PCR, the ideal cut-off for admission samples was lower in the range of 0.25–0.5 with sensitivity, specificity and AUROCC for ELISA OD at ≥0.25 of 75.0%, 72.6%, and 0.45.

The best compromise for the ELISA OD cut-off for scrub typhus diagnosis with IgG antibodies was higher than for IgM and likely to be in the range of 1.2–1.8 for admission, follow-up and combined samples with IFA. With sensitivity, specificity and AUROCC for ELISA OD at ≥1.50 of 82.4%, 81.2%, and 0.82 for admission, 81.8%, 86.1%, and 0.84 for follow-up and of 66.2%, 84.9%, and 0.76 for combined samples. Whilst for PCR, the ideal cut-off for admission samples was lower in the range of 0.25–0.5 with sensitivity, specificity and AUROCC for ELISA OD at ≥0.25 of 60.0%, 66.0%, and 0.63.

### ELISA OD cut-off for sero-epidemiological screening for IgG antibodies with IFA confirmation

To use ELISA as a screening tool prior to IFA confirmation for sero-epidemiological purposes, a conservative IFA titer of ≥1:100 would need to be detected with high sensitivity. A cut-off OD of 0.5 would provide a sensitivity of 96.4% (95% Confidence Interval (CI): 93.8%-98.1%) and a specificity of 46.9% (95%CI: 40.0–53.9%) for IgG admission antibodies to detect an IFA titer of ≥1:100.

## Discussion

We investigated the diagnostic accuracy of the novel in-house STG ELISA and showed a range of potential cut-off ODs for IgM and IgG antibodies for its use as a screening or diagnostic tool of scrub typhus infection.

A strong association was found between ELISA OD values and IFA reciprocal titers for admission (IgM: Spearman *r*^2^, 0.70; IgG Spearman *r*^2^, 0.76) and follow-up sera (both IgM and IgG: Spearman *r*^2^, 0.76). Previous studies suggest that the performance of InBios Scrub Typhus IgM ELISA kit is equal to IFA IgM [21] or better than the IFA [16]. Since the correlation in our results is comparable to that observed in previous studies for InBios Scrub Typhus Detect IgM ELISA (Spearman *r*^2^, 0.68) [[11] it suggests that the diagnostic accuracy of the MORU in-house STG ELISA is likely comparable to the IFA and to the InBios Scrub Typhus Detect ELISA, and also validates the use of the in-house STG ELISA as a reference standard. Future research could compare the diagnostic performance of the MORU in-house STG ELISA with commercially available ELISA tests.

The optimal ELISA cut-off will depend on the planned use of the test. For initial background antibody screening in a seroepidemiology study, with a subsequent “gold-standard” IFA to determine the antibody titer for those positive by ELISA, increased sensitivity would be more desirable and specificity may be compromised [10,11]. The appropriate cut-off would likely be ~0.5 OD for IgG, since we found a sensitivity of 96.4% to detect an IFA titer ≥1:100. If the ELISA is to be used as a stand-alone diagnostic test, without confirmation by IFA and/or PCR, an optimal compromise between sensitivity and specificity would likely be around 1.0 for IgM and a higher cut-off of 1.5 for IgG. The higher cut-off for IgG antibodies could reflect a higher background level of IgG antibodies compared to IgM antibodies due to longer persistence of IgG antibodies compared to IgM for scrub typhus after infection [22]. The cut-off values attained in this study were higher than those used in many other Southeast Asian endemic regions [11,18,19,23]. The selection of an absolute cut-off is dependent on the practical application and the geographical specific endemicity. One study conducted in Northern Thailand reported cut-offs between 0.3–0.6 for IgM [11], while another study reported cut-offs between 1.8–2.2 for IgM [24]. The cut-offs applied in India varied from 0.5–1.0 for IgM and was set at 1.8 for IgG [15,25]. Studies using the InBios Scrub Typhus ELISA kits tend to apply a cut-off of 0.5 OD, as the kit provides control serum samples without regard to the geographical location where the study is being conducted [17]. This poses a challenge, since the cut-off generated in one study would not necessarily be useful in different geographic areas with varying levels of background immunity. This highlights the need for further regional assessment of background immunity and the testing of new serological tests depending on in which community the test is to be used.

Karp, Kato, Gilliam and TA716 pooled antigens were used in the MORU in-house STG ELISA. Studies have identified Karp and Gilliam strains circulating in Thailand and Laos, with predominantly Gilliam-type strains in Laos [26]. New genotypes have been described in Laos that may be associated with unrecognised type strains [27]. The diversity of strains isolated from Laos may limit the sensitivity and specificity of serological tests that do not include these strains [26,27]. It would be desirable to further characterize the antigenic strains in Laos, and other geographic areas endemic to scrub typhus, since this would inform researchers and healthcare workers about the potential limitations of diagnostic tests due to the usage of a restricted range of strains. Such local variation in antigenic composition is likely to contribute to discrepancies in assay sensitivity and specificity [10]. Furthermore, further studies are required to determine the potential cross-reactivity with novel Orientia spp. such as *O. chuto* [28].

There were several limitations in this study. Firstly, the participants were relatively young with a median age of 22 years. Scrub typhus background antibody titers are likely to increase with age, due to (repeated) exposure, which could have led to an overall lower background level of antibodies than in the general population in Laos. This could potentially influence the ELISA OD cut-off level, since a higher background antibody level could lead to a higher ELISA OD cut-off to be needed for an older population. Secondly, only a limited number of samples tested positive with PCR at the admission samples making it difficult to properly study the association between ELISA and PCR results. This could have been because PCR is only able to detect *O*. *tsutsugamushi* in the blood of some participants due to a low bacterial blood density of scrub typhus [29]. The much lower ELISA cut-offs for PCR positive participants compared to IFA positive participants could potentially be explained by the high specificity but low sensitivity of PCR. Thirdly, a larger sample size with more positive samples would have allowed better estimations of the optimal cut-offs and improved the overall applicability of the observed results. Further studies are needed to investigate the use of the MORU in-house STG ELISA cut-offs in other endemic regions to be able to consider background antibody levels for the determination of a cut-off [17].

We have shown that the MORU in-house STG ELISA is an accurate diagnostic test compared to the imperfect IFA “gold-standard” and can be used for initial screening in seroepidemiology studies with an ELISA OD cut-off of 0.5 and potentially as a stand-alone diagnostic test instead of IFA. The optimal diagnostic cut-off would depend on the use of the test as either a diagnostic tool or for cross-sectional screening. Therefore, these cut-offs would need to be adjusted to find an appropriate compromise between sensitivity and specificity. Considering that ELISAs are less expensive, easier to use, standardised and automated [24], they have the potential to replace the imperfect “gold standard” IFA. ELISAs could likely be suitable as a stand-alone test in low-resource settings where no IFA and PCR assays are available, improving the rate of scrub typhus diagnoses and aiding in reducing the burden of scrub typhus morbidity and mortality.

## Supporting information

S1 Fig Frequency distribution of ELISA optical densities (OD)s for admission and follow-up samples for IgM (A) and IgG (B). Bin width: 0.05 OD.
(PNG)

S2 Fig Distribution of admission and follow-up sample IFA titers for IgM (A) and IgG (B) with an ELISA OD≥0.5.
(TIFF)

S1 Table Number of IFA titers for admission and follow-up IgM and IgG samples with an ELISA Optical Density (OD)<0.5. (PDF)

S2 Table Number of IFA titers for admission and follow-up IgM and IgG samples with ELISA Optical Density (OD)≥0.5. (PDF)

S3 Table Participant characteristics and ELISA with IFA results for participants with a positive PCR test for scrub typhus. (PDF)
