# Supplementary figures and images for: Diagnostic accuracy of an in-house Scrub Typhus enzyme linked immunoassay for the detection of IgM and IgG antibodies in Laos
Source: PLoS Negl Trop Dis. 2020 Dec 7;14(12):e0008858. doi: 10.1371/journal.pntd.0008858 (PMC7746293; doi:10.1371/journal.pntd.0008858)

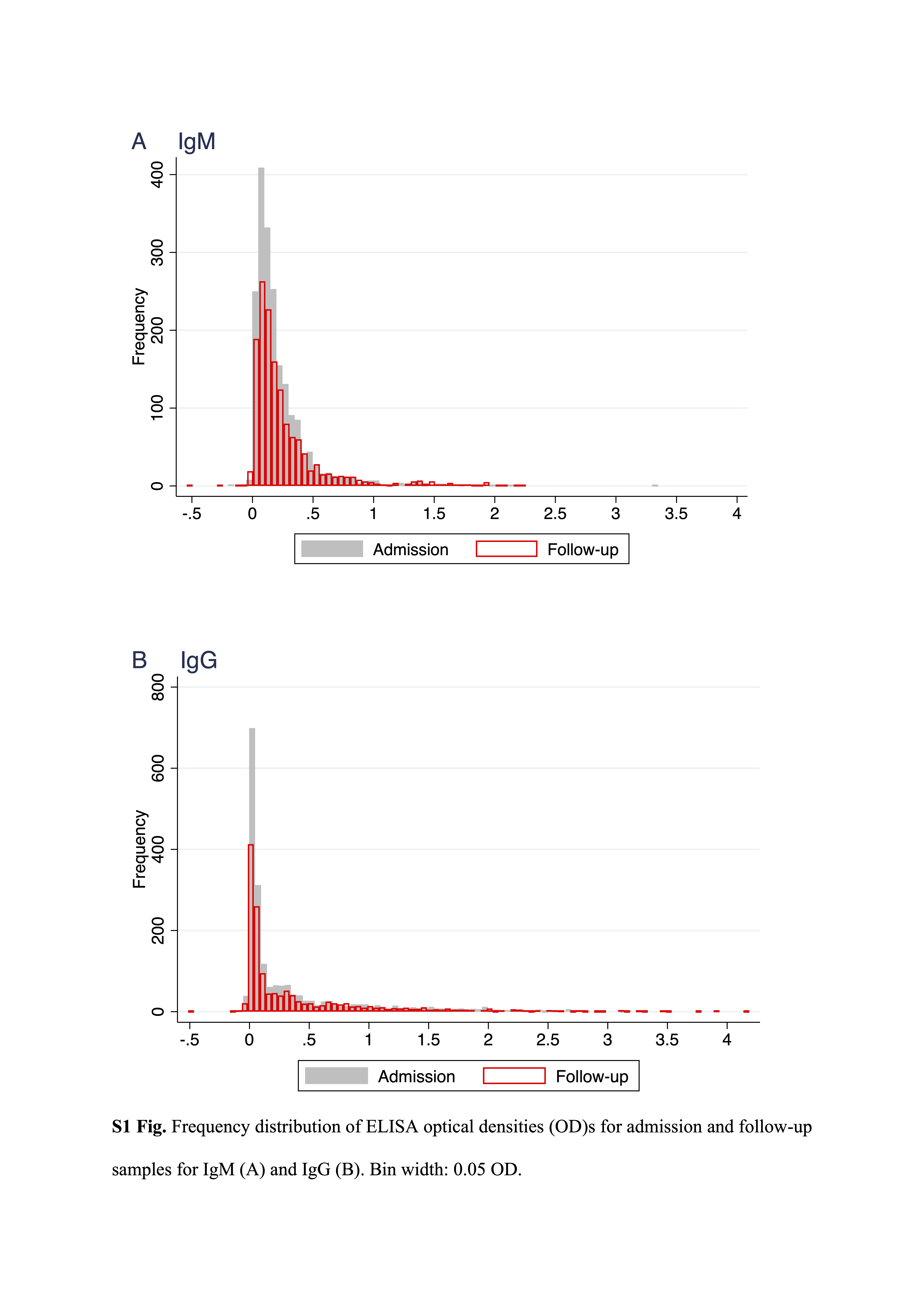

Supplement: S1 Fig — Frequency distribution of ELISA optical densities (OD)s for admission and follow-up samples for IgM (A) and IgG (B). Bin width: 0.05 OD. (PNG) [file pntd.0008858.s001.png]

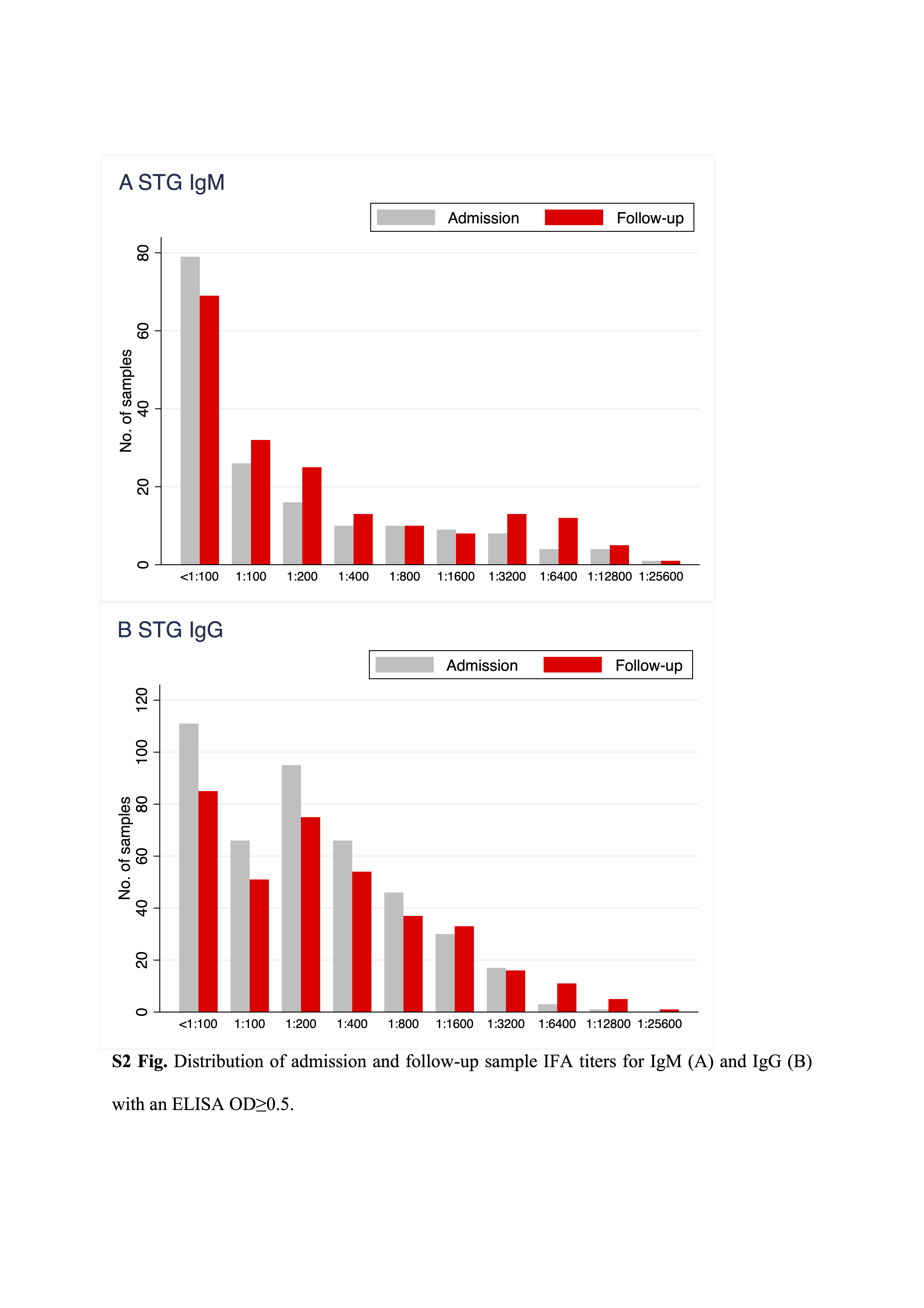

Supplement: S2 Fig — Distribution of admission and follow-up sample IFA titers for IgM (A) and IgG (B) with an ELISA OD≥0.5. (TIFF) [file pntd.0008858.s002.tiff]
