# Supplementary material for: Diagnostic accuracy of an in-house Scrub Typhus enzyme linked immunoassay for the detection of IgM and IgG antibodies in Laos
Source: PLoS Negl Trop Dis. 2020 Dec 7;14(12):e0008858. doi: 10.1371/journal.pntd.0008858 (PMC7746293; doi:10.1371/journal.pntd.0008858)
Supplement: S1 Table — (PDF) [file pntd.0008858.s003.pdf]

Supplementary Table 1. Number of IFA titers for admission and follow-up IgM and IgG samples with an ELISA Optical Density (OD)<0.5.

IFA titer	Adm IgM	FU IgM	Adm IgG	FU IgG
<1:100	140	86	98	82
1:100	2	2	5	3
1:200	1	0	3	1
1:400	0	0	1	0
1:800	0	0	3	0
≥1:1600	0	0	0	0
Total*	143	88	110	86

* The IFA titer was determined for both the admission and follow-up sample for an antibody isotype if either the admission or follow-up ELISA OD≥0.50 for that antibody isotype. For one participant with an IgM ELISA OD<0.50 for both admission and follow-up sample, the IFA titers for IgM had been determined for unknown reasons but was included in the analysis. To retrospectively validate the ELISA OD cut-off of 0.50, a further 50 samples with ELISA OD results of <0.50 for both admission and follow-up IgM and IgG were retested for both IgM and IgG IFA titers. Adm=admission; FU=follow-up.
