# Supplementary material for: Diagnostic accuracy of an in-house Scrub Typhus enzyme linked immunoassay for the detection of IgM and IgG antibodies in Laos
Source: PLoS Negl Trop Dis. 2020 Dec 7;14(12):e0008858. doi: 10.1371/journal.pntd.0008858 (PMC7746293; doi:10.1371/journal.pntd.0008858)
Supplement: S2 Table — (PDF) [file pntd.0008858.s004.pdf]

Supplementary Table 2. Number of IFA titers for admission and follow-up IgM and IgG samples with ELISA Optical Density (OD)≥0.5

IFA titer	Adm IgM	FU IgM	Adm IgG	FU IgG
<1:100	79	69	111	85
1:100	26	32	66	51
1:200	16	25	95	75
1:400	10	13	66	54
1:800	10	10	46	37
1:1600	9	8	30	33
1:3200	8	13	17	16
1:6400	4	12	3	11
1:12800	4	5	0	5
1:25600	1	1	1	1
	167	188	435	368

Adm=admission; FU=follow-up.
