# Supplementary material for: Diagnostic accuracy of an in-house Scrub Typhus enzyme linked immunoassay for the detection of IgM and IgG antibodies in Laos
Source: PLoS Negl Trop Dis. 2020 Dec 7;14(12):e0008858. doi: 10.1371/journal.pntd.0008858 (PMC7746293; doi:10.1371/journal.pntd.0008858)
Supplement: S3 Table — (PDF) [file pntd.0008858.s005.pdf]

Supplementary Table 3. Participant characteristics and ELISA with IFA results for participants with a positive PCR test for scrub typhus.

				Admission		Follow-up		Admission		Follow-up	
Gender	Age (years)	Days of illness	Days between samples	IgM ELISA	IgM IFA	IgM ELISA	IgM IFA	IgG ELISA	IgG IFA	IgG ELISA	IgG IFA
Male	15	14	-	0.3453				1.5233	1:400		
Female	6	7	15	0.3561	<1:100	1.3800	1:3200	0.0163		0.4568	
Female	28	7	-	0.3263				0.0537			
Female	24	5	14	0.1567	<1:100	0.8209	1:3200	0.5630	<1:100	1.9878	1:1600
Female	15	5	13	0.6495	1:100	1.4124	1:800	0.0442		0.3597	
Female	23	3	11	0.8341	1:100	1.8167	1:3200	0.1671	<1:100	0.9227	1:800
Male	20	5	-	0.1209				0.9244	1:100		
Male	36	6	20	0.1031		0.1886		1.6316	1:400	1.6039	1:1600
Female	21	2	34	0.3842	<1:100	1.6388	1:6400	0.0396	<1:100	0.6114	1:3200
Male	39	7	33	0.2249		0.2302		2.6624	1:800	3.9012	1:12800
Female	23	3	14	0.2667		0.3316		1.0355	<1:100	3.1153	1:800
Male	15	8	6	0.3591		0.3143		2.0702	1:400	3.7655	1:6400
Male	4	3	34	0.2259	<1:100	1.1886	1:800	0.0198	<1:100	1.0788	1:400
Female	7	4	24	1.3137	1:1600	1.3700	≥1:25600	0.1958	<1:100	0.9882	1:200
Male	7	14	11	2.1602	1:3200	2.2157	1:6400	0.4460	<1:100	0.6859	<1:100
Male	28	10	-	1.6257	≥1:25600			0.2856			
Male	47	14	3	0.5662	<1:100	0.5548	<1:100	2.2096	1:6400	1.6814	1:6400
Male	30	7	-	0.9457	1:6400			0.5200	1:200		
Female	30	16	17	1.3039	1:3200	1.4751	1:3200	0.6423	1:100	1.0462	1:200
Female	3	9	12	1.0240	1:100	1.3653	1:400	0.0676		0.2447	

Red colour indicates an ELISA OD≥0.5 or IFA IgM ≥1:3,200 at admission or IgM ≥1:3,200 at follow-up with four-fold rise compared to admission; IgG ≥1:1,600 at admission or IgG ≥1:1,600 at follow-up with four-fold rise compared to admission.
